# Planning, implementation and revision of the longitudinal scientific curriculum at the Medical School Brandenburg

**DOI:** 10.3205/zma001671

**Published:** 2024-04-15

**Authors:** Julia Schendzielorz, Philipp Jaehn, Christine Holmberg

**Affiliations:** 1 Brandenburg Medical School Theodor Fontane, Center for Curriculum Development and Educational Research, Brandenburg a.d.H., Germany; 2 Brandenburg Medical School Theodor Fontane, Institute of Social Medicine and Epidemiology, Brandenburg a.d.H., Germany; 3 Joint Faculty of the Brandenburg University of Technology Cottbus-Senftenberg, Brandenburg Medical School Theodor Fontane and the University of Potsdam, Faculty of Health Sciences, Potsdam, Germany

**Keywords:** competency-based education, scientific competencies, faculty development, evidence-based medicine, curriculum development

## Abstract

**Objectives:**

The aim of this paper is to present the development of a longitudinal curriculum for medical students that is rooted in the particularity of the medical sciences and that aims to build and strengthen medical students’ scientific skills and use thereof in clinical practice.

**Methods:**

The curriculum development was initiated based on students’ feedback on the initial curriculum. To improve and expand the curriculum appropriately, a needs assessment, a literature review to define science specific to the medical sciences and practice, and an analysis of national and international curricula were performed. The curriculum development followed the PDCA cycle (Plan-Do-Check-Act).

**Results:**

The curriculum extends across the entire medical study programme from semesters 1 to 10. It consists of the seminar series on basic conduct and the epistemological groundings of science, scientific methods in medical research and health sciences, statistics and the scientific internship. Up to the sixth semester, the focus is on the acquisition of skills and abilities to work on and carry out a concrete research project; starting in semester seven, the critical evaluation and application of research results in everyday clinical practice are introduced. The curriculum is taught by epidemiologists, anthropologists, statisticians and public health scholars. Starting in semester seven, seminars are generally taught together with clinicians as tandem teaching. The curriculum is regularly assessed and adjusted.

**Conclusions:**

The Brandenburg Scientific Curriculum can be seen as a model of a longitudinal curriculum to teach scientific thinking and acting. One that is at the same time highly integrated in the medical curriculum overall. A central coordination point seems to be necessary to coordinate the teaching content and to ensure that teachers are interconnected. Furthermore, a complex curriculum in scientific methodology requires a set of teachers from a range of disciplinary backgrounds. To ensure equally high-quality education, the variability of research projects and faculty must be taken into account by establishing generally applicable evaluation criteria and fostering faculty development, and providing all students supporting courses throughout the research project.

## Zusammenfassung

**Zielsetzung:**

Ziel dieses Beitrags ist die Entwicklung eines longitudinalen Curriculums für Medizinstudierende vorzustellen, das darauf abzielt, die wissenschaftlichen Kompetenzen der Medizinstudierenden zu entwickeln und zu stärken und diese in der klinischen Praxis unter Berücksichtigung der Besonderheiten der medizinischen Wissenschaften anzuwenden.

**Methoden:**

Die Entwicklung des Curriculums wurde auf der Grundlage des Feedbacks der Studierenden zum ursprünglichen Curriculum eingeleitet. Um das Curriculum entsprechend zu verbessern und zu erweitern, wurden eine Bedarfsanalyse, eine Literaturrecherche zur Definition von medizinischer Wissenschaft und Praxis sowie eine Analyse nationaler und internationaler Curricula durchgeführt. Die Entwicklung des Curriculums folgte dem PDCA-Zyklus (Plan-Do-Check-Act).

**Ergebnisse:**

Das Curriculum erstreckt sich über das gesamte Medizinstudium vom ersten bis zum zehnten Semester und besteht aus den Seminarreihen zu den Grundlagen der Wissenschaft und Erkenntnistheorie sowie Methoden Wissenschaftlichen Arbeitens und den Modulen Biometrie sowie Wissenschaftspraktikum. Bis zum sechsten Semester steht der Erwerb von Fähigkeiten und Fertigkeiten zur Bearbeitung und Durchführung eines konkreten Forschungsprojektes im Vordergrund, ab dem siebten Semester wird in die kritische Bewertung und Anwendung von Forschungsergebnissen im klinischen Alltag eingeführt. Das Curriculum wird von Epidemiolog*innen, Anthropolog*innen, Statistiker*innen und Public Health Wissenschaftler*innen unterrichtet. Ab dem siebten Semester werden die Seminare in der Regel gemeinsam mit Kliniker*innen im Tandem unterrichtet. Das Curriculum wird regelmäßig evaluiert und angepasst.

**Schlussfolgerung:**

Das Brandenburger Wissenschaftscurriculum kann als Modell eines longitudinalen Curriculums zur Vermittlung des wissenschaftlichen Denkens und Handelns angesehen werden. Ein Curriculum, das gleichzeitig in hohem Maße in das medizinische Curriculum insgesamt integriert ist. Eine zentrale Koordinationsstelle erscheint notwendig, um die Lehrinhalte abzustimmen und die Vernetzung der Lehrenden zu gewährleisten. Darüber hinaus erfordert ein komplexes Curriculum in wissenschaftlicher Methodik eine Reihe von Lehrenden mit unterschiedlichem disziplinärem Hintergrund. Um eine qualitativ hochwertige Ausbildung zu gewährleisten, muss die Variabilität der Forschungsprojekte und der Lehrenden berücksichtigt werden.

## Introduction

Medicine is considered both an “art and a science” [1]. This statement represents the tension in which modern scientific medicine stands between the knowledge developed by the sciences and the particularity of treating individual patients [2]. Medicine is always oriented toward the individual patient and thus scientific knowledge is applied, or better “tinkered with”, through individual care-related medical decision-making and care-giving. Medical education seeks to “harness universal and experimental scientific knowledge within an individual and interpretative framework” [3]. Today the skills necessary for good doctoring are referred to as clinical reasoning and encompass the ability to integrate biomedical/epidemiological knowledge and historical/hermeneutic understanding with common sense knowledge knowing and medical theory [4]. Thus, medical knowing and medical practice consist of a range of different forms of knowledge [3]. These particularities of medicine necessitate knowledge and understanding from the natural sciences, social sciences and humanities [5], [6], [7], all of which need to be taught in medical education. 

In Germany in recent years, scientific thinking and argumentation has come into focus in medical education as one of the core competencies that science-based education needs to enable [8], and which requires more attention in medical education. The call has been put forth to strengthen the science-based and practical research competencies of medical students. This call has also been taken up in the drafted revision of the Medical Licensing Regulations (AP), which are currently under negotiation, in which the integration of a longitudinal scientific curriculum is being implemented [9]. However, only a few longitudinal curricula are known that teach a combination of basic science principles and their integration in clinical practice throughout the entire medical degree [10], [11] programme. 

Therefore, in this article we present the development of a longitudinal scientific curriculum for medical students that is rooted in the particularity of the medical sciences and that aims to build and strengthen medical students’ scientific skills and use thereof in clinical practice. 

For the development of such a curriculum, the first question to be addressed is how the plurality of scientific disciplines necessary to practice medicine today can be represented in a scientific curriculum. The second question is that of scientific reasoning itself: What can be understood as scientific reasoning within a medical scientific context? 

Over the past 30 years, the concept of evidence-based medicine (EBM) has gained increasing momentum, and in this concept some competencies necessary for medical practitioners today have been specified and curricula developed. EBM aims to support the building of medical practice on epidemiological study findings and on the skills of physicians and their patients [12]. For the successful application of research results in everyday clinical decision-making, basic principles of clinical epidemiology and biometry, as well as skills in searching, understanding, analysing, interpreting and critically evaluating research results from a wide variety of disciplines, are a necessary component. Universities around the world have developed EBM curricula with different formats, durations and frequencies [13], [14]. The goals are the acquisition of key qualifications, such as acquiring new information and relating it to previous knowledge, but also the development of problem-solving skills and a better linking of theoretical and experiential knowledge. This kind of learning is perceived to enrich clinical experience, as well as provide a better understanding of the basic science disciplines [15].

In Germany, recommendations have been put forward to include such skills in general medical training [16], [17]. Standards have been set accordingly in the National Competence-Based Learning Objectives Catalogue for Medical Education (NKLM) [https://nklm.de/zend/menu]. The NKLM serves as a non-binding orientation aid for competence-based teaching at German medical faculties; however, binding implementation is intended only for the upcoming revision of the Medical Licensing Regulations (AO). A range of German medical faculties have already implemented corresponding teaching content in different scopes and formats, or are in the planning stage of doing so [18], [19]. The spectrum ranges from four to 20 weeks in duration, and may be compulsory for all students [20], [21], [22], [23], part of an individual focus over several semesters [23], [24], or an optional curricular element [25], [26]. Such approaches present an important starting point to develop a comprehensive longitudinal scientific curriculum that fits the particularities of the medical sciences and practice.

Science as we understand it stands for “teachable knowledge” [3]. It thus encompasses the natural and social sciences as much as the humanities. A scientific curriculum therefore has to define how these scientific approaches will be included. Only then, we would argue, can students adequately obtain the various skills necessary for clinical reasoning.

## Methods used for the curriculum development

**Figure 1:**
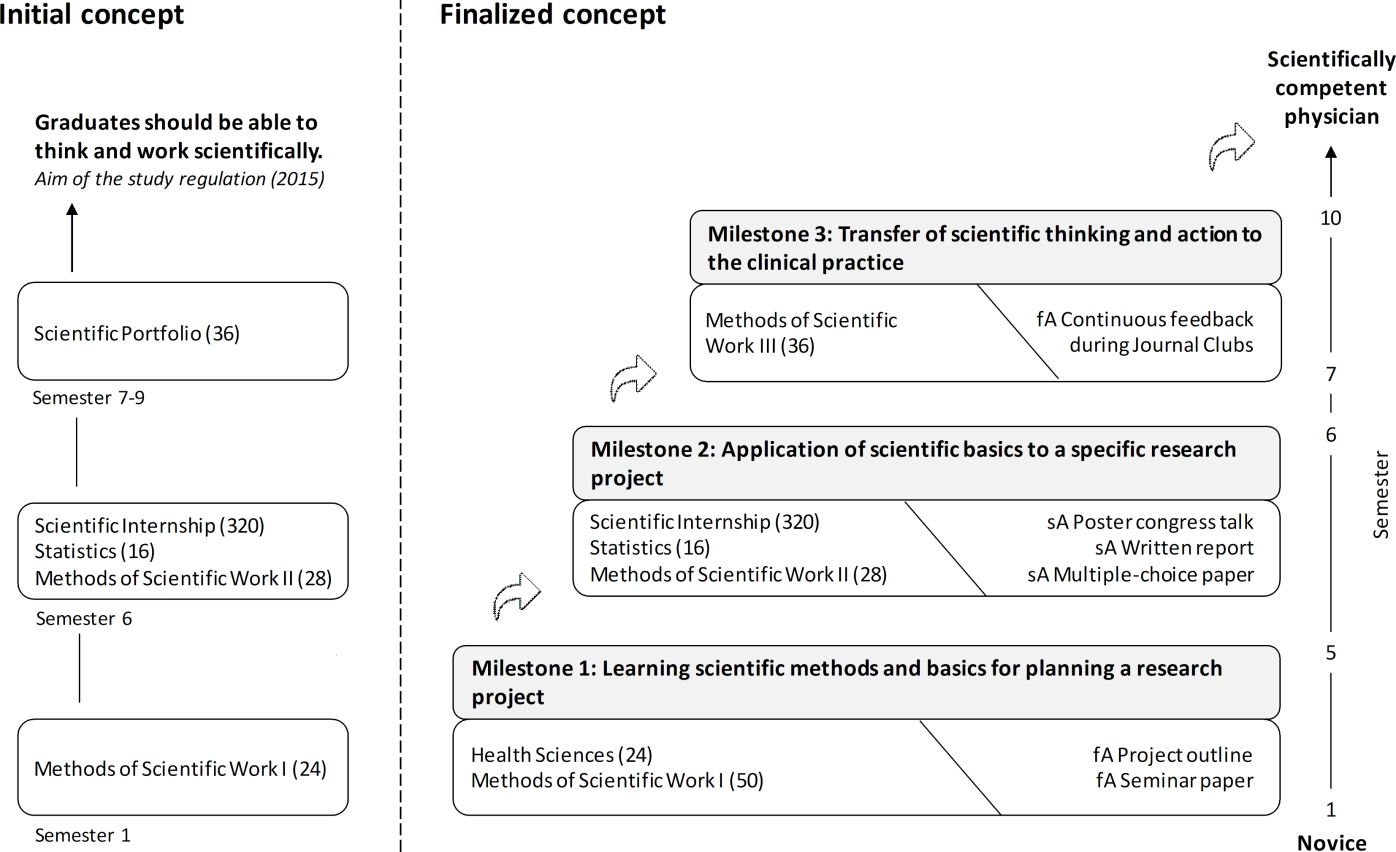
Elements and teaching units (numbers in brackets) of the Brandenburg Scientific Curriculum in the original (left) and in the currently practiced concept with its milestones and formative (fA) and summative (sA) assessment formats (right)

The initial scientific curriculum of the Brandenburg Reformed Medical Study Programme (BMM) consisted of a seminar series Methods of Scientific Work I (MSW I) in the first semester with 24 teaching units (TU), MSW II in the sixth semester with 28 TU, and a scientific portfolio between the seventh and ninth semesters with 36 TU, as well as the one-week module Statistics (ST) and the eight-week module Scientific Internship (SI), both taught in the sixth semester (see figure 1 (Fig. 1)). Since the SI is a milestone in the scientific-methodological training of the students, a student evaluation took place after completion of the module, focusing not only on general satisfaction with the SI, but also on how well the students felt prepared for the SI through MSW I and MSW II. 

Participation was voluntary and anonymous. The survey consisted of four closed questions with a 5-point Likert scale. Subsequently, three categories were formed from the five levels of the Likert scale. Levels one and two were aggregated into the category “disagree”, level three into the category “neither”, and levels four and five into the category “agree”. In addition, the students were given the opportunity to comment on any suggestions or ideas for improvement. The free text comments were first coded inductively in a thematic analysis, systematized into categories, and the number of mentions within the categories quantified [27].

After the first cohorts of medical students had completed and evaluated the MSW seminar series I and II and the ST and SI modules, we used the PDCA cycle (Plan-Do-Check-Act) to expand and finalize the conceptual development of the Brandenburg Scientific Curriculum (BraWiC). This is a method of continuous process improvement and is applied in business, health and education [28]. It is composed of four steps; the transfer to curriculum development is italicised in brackets.

1. Plan: Define a problem and make a hypothesis about possible causes. Develop specific goals (*competencies*) and alternative concepts (define* new content, develop new teaching events and formats*).
2. Do: Implementation of the previously developed concept (*new curriculum*) and continuous monitoring (*evaluation through written/oral feedback*).
3. Check: Analysis of evaluation results and identification of areas for improvement, if necessary.
4. Act: Determination of further steps based on the findings, i.e. standardisation or further revision of the concept (*implementation of the curriculum or further revision*).

In a last step the learning content of the BraWiC was compared with the learning objectives (LO) of the NKLM 2.0 [https://nklm.de/zend/menu] to ensure that the required skills and knowledge are covered and that the training meets the national standards. 

## Results

In the following, the steps of the PDCA cycle, as they were employed to expand and finalize the BraWiC, are explained in more detail.

### Step 1 – plan

#### 1. Problem definition 

The starting point for the curriculum expansion and further development was a review of the NKLM, existing scientific curricula, and the student evaluations given by the first cohorts of the BMM following the SI. 

**Figure 2:**
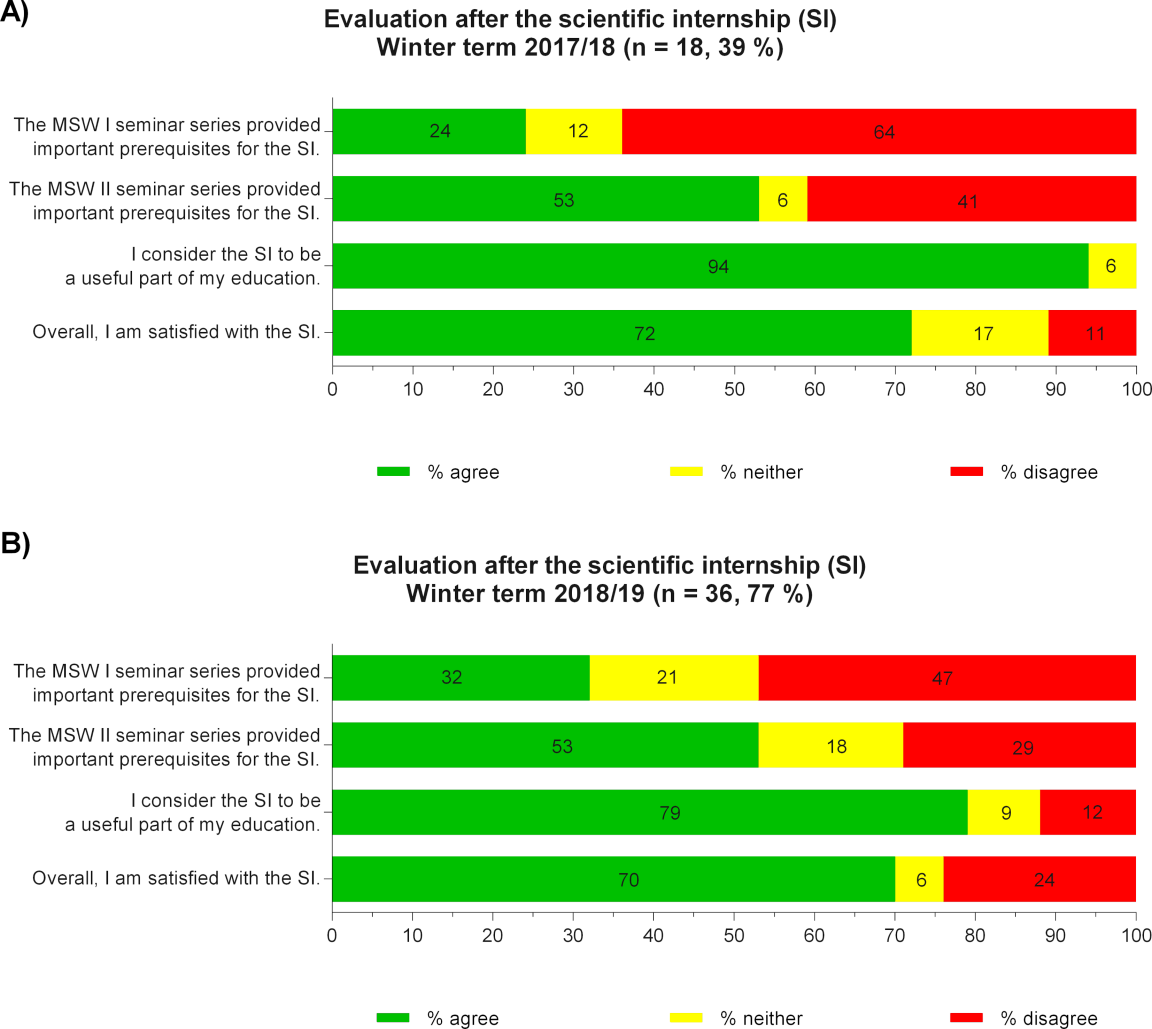
Evaluation results of the first (A) and second (B) cohorts after completion of the scientific internship (SI)

Response rates from the student evaluations were 39% (n=18) for the first cohort and 77% (n=36) for the second cohort. For MSW I, only 24% (1^st^ cohort) and 32% (2_nd_ cohort) of participating students agreed with the statement that important prerequisites for the SI were taught (see figure 2 (Fig. 2)). Analysis of the free-text comments resulted in a clustering of responses into six categories (see table 1 (Tab. 1)). The majority of the responses were related to statistics and the desire to expand preparatory courses. MSW II was rated positively by half of the students (53% each, see figure 2 (Fig. 2)), and a large majority also agreed that the SI was a useful part of the degree program (1^st^ cohort: 94%, 2^nd^ cohort: 79%). Overall, there was a high level of satisfaction with the SI (1^st^ cohort: 72%, 2^nd^ cohort: 70%). 

#### 2. Hypothesis formulation

From the analysis of the feedback received, the hypothesis was formulated that the preparation for the SI through the accompanying seminars and modules was inadequate. This related to the content to be taught, the scope of the teaching, but also the timing and organizational aspects.

#### 3. Defining specific goals

In order to achieve the goal of realising a comprehensive education for future doctors in scientific thinking and acting, the following overarching competencies were formulated for the BraWiC by the authors. The graduate will be able to:

1. Find relevant medical literature and use it for the adequate treatment of a specific patient.
2. Describe the rules and procedures of clinical practice guideline development.
3. Identify the guidelines needed for a guideline-based treatment of a patient and assess their relevance for the individual patient.
4. Interpret and evaluate scientific research results.
5. Work on a medical research question under guidance and write a scientific paper.

In addition, three milestones were defined for the BraWiC:

1. Semester 1-5: Learning scientific methods and basics for planning a research project.
2. Semester 6: Application of scientific basics to a specific research project. In the process, learning new skills such as poster design, abstract writing.
3. Semester 7-10: Transfer of scientific thinking and action to the clinical practice.

#### 4. Concept development

The initial curriculum was expanded to promote the acquisition of the higher-level competencies mentioned in paragraph 3. above. Building on the LO already available for the ST and SI modules, the particular aim was to improve preparation for the SI and to conceptualize the curriculum for semesters 7 to 10. The following documents were reviewed to determine the content: Chapter 14a “Medical Scientific Skills” of the NKLM 1.0 [29], the curriculum “Evidence-based decision-making” of the German Network for EBM (Deutsches Netzwerk Evidenzbasierte Medizin e.V.) [30], as well as the modules “Basic Epidemiology” of the London School of Hygiene and Tropical Medicine and “Research for Medicine and Health” of the Medical Faculty of the University of Southampton. Subsequently, an expanded curriculum proposal was developed by JS and completed and finalized in discussion sessions with all authors. 

The main measures of further development included a complete restructuring of the MSW curriculum. To this end, teaching content was shifted to earlier semesters (e.g. study designs for clinical research, descriptive statistics and research ethics) and additional content with a scope of 26 TU was integrated. Essential new elements were the exercises in formulating a research question as well as the introduction of a written project outline. The aim of these changes is to deal with the research topic and the project requirements at an early stage, but also to put students in contact with supervisors. Furthermore, the cooperation with the ethics committee was intensified and a deadline was set for submitting an ethics application. This is to ensure there is sufficient time for any corrections before the regular start of the internship. In addition, the focus on medical-ethical issues in the context of medical research was intensified and the methodological diversity of medical research was particularly addressed. The methodological content of theoretical subjects and empirical social research were therefore integrated alongside seminars on clinical-epidemiological methods and statistics. 

#### 5. Concept description

The BraWiC consists of the seminar series MSW I to III and Health Sciences (HS), as well as the modules ST and SI (see figure 1 (Fig. 1)). 

#### Methods of Scientific Work (MSW)

The curricular bracket of the BraWiC is formed by the MSW, which extends over the entire degree programme with a scope of 114 TU and is divided into three phases (MSW I to III, see attachment 1 , table S3). From the first to the fifth semester, students learn basic scientific methods and the fundamentals of planning a scientific research project (MSW I). In the sense of arriving and learning to navigate everyday university life, introductory seminars are given on time management, learning and working techniques, as well as literature research. Students write their first seminar paper, which focuses on the form and structure of academic texts and is assessed with structured feedback. Study designs of clinical research, systematic errors, qualitative research and research ethics are addressed in further courses in MSW I. Furthermore, exercises on formulating a research question and designing a concrete scientific project are offered. The focus of MSW II is on the acquisition of skills and abilities to undertake the SI and includes an in-depth literature study as well as an introduction to study and data management. In addition, students are trained in data analysis using statistical software, but also in writing a scientific paper, creating a conference poster and writing a conference abstract. In small group teaching, students can share experiences, challenges and strategies in the implementation of their own projects or they can discuss the poster preparation and presentation for the research project conference. In addition, there are seminars on health care research and translational research, as well as on opportunities for a career in medical research.

The transition from organ-based learning to the primarily clinical-practical study section takes place in the seventh semester. This means that the application of scientific principles and findings including scientific reasoning in and for everyday clinical practice is the focus of the MSW content until the end of the degree programme (MSW III). In the seventh semester, the development and application of clinical practice guidelines are addressed and the transferability of study results to clinical practice is critically reflected upon. Special attention is paid to the link between EBM and the active involvement of the patient in the medical decision-making process by means of case studies and practical exercises, as well as in the teaching format Teamwork, Reflection, Interaction and Communication (TRIK). Next to this, critical reading and evaluation of medical studies and the application of evaluation schemes are addressed in order to introduce the teaching format of the Journal Club (JC). Between the eighth and tenth semesters, one to two JC are held per clinical module, in which a scientific article on a topic relevant to the current module is discussed. The discussion is focussed on the methodological strengths and limitations of clinical studies in order to evaluate the applicability of the results in practice. To strengthen clinical reasoning based on scientific studies the JC is taught jointly by a methodologist and a clinician.

#### Health sciences (HS)

After an introduction to the philosophy of science and scientific work in MSW I (2 TU) in the first semester, the basics of population-based and social science research methods are introduced in HS in the second semester. Students gain initial insights into the interpretation of epidemiological measures, basic principles of population-based research methods in health sciences and the basics of qualitative social research. These contents prepare for further basic education in the context of MSW I in semesters three and four.

#### Statistics (ST)

The SI is preceded by the one-week module ST with a scope of 16 TU. Aspects of clinical-epidemiological study designs and systematic errors are repeated and deepened, and the topics of descriptive and inferential statistics are introduced in lectures, seminars and guided exercises in order to create a basis for independent data analysis in the SI. The students are assessed by a multiple-choice paper at the end of the semester.

#### Scientific internship (SI) 

The SI takes place for a total of eight weeks on a full-day basis and consists of 320 TU. The aim is to apply the skills acquired in MSW I and II to a concrete research project and to learn working methods necessary to address a research question, which include in particular the critical reading of scientific literature. Students select their SI from a project database during the 5th semester prior to the SI. Lecturers and researchers associated with the medical school offer SI projects and are the supervisor during the internship. Supervisors are regularly offered training by the university to introduce them to the scientific standards taught to the students and to familiarize them with the LO of the SI. With the support of the MSW seminars in the 5th semester, students contact their respective supervisors and develop a research question for their project. The project can be carried out in an inpatient or outpatient clinic, a theoretical institute or a research institution. A preliminary project description has to be submitted by the students at the beginning of semester 6 and is presented at the MSW prior to the start of the SI. The intertwining of SI and MSW is important to provide support from the university to students and supervisors to ensure the standards are taught in the BraWiC. During the internship, students have to complete a laboratory notebook, which forms the basis for the weekly meetings with the supervisor. A project report is submitted and reviewed by the supervisor. In addition, a conference abstract and scientific poster are presented at an internal university congress to a review panel. Standardised evaluation sheets are used to evaluate the project report by the supervisor and the poster presentation by the review panel. 

### Step 2 – do

To implement the curriculum, individual discussions were held with the respective lecturers on the content and didactic design of the new teaching sessions (e.g. biostatistics, biochemistry). However, as a large part of the newly designed sessions pertained to population-based and social sciences and were assigned to the Chair of Social Medicine and Epidemiology (CH), most of the discussions took place within the group of authors. From 2019, the revised curriculum was gradually integrated into current study semesters (third and fifth) or from the beginning of the study program. Continuous feedback rounds were held with lecturers and students. In addition, the overall student evaluation of the SI, MSW I and II a written survey was conducted as before.

### Steps 3 & 4 – check & act 

**Figure 3:**
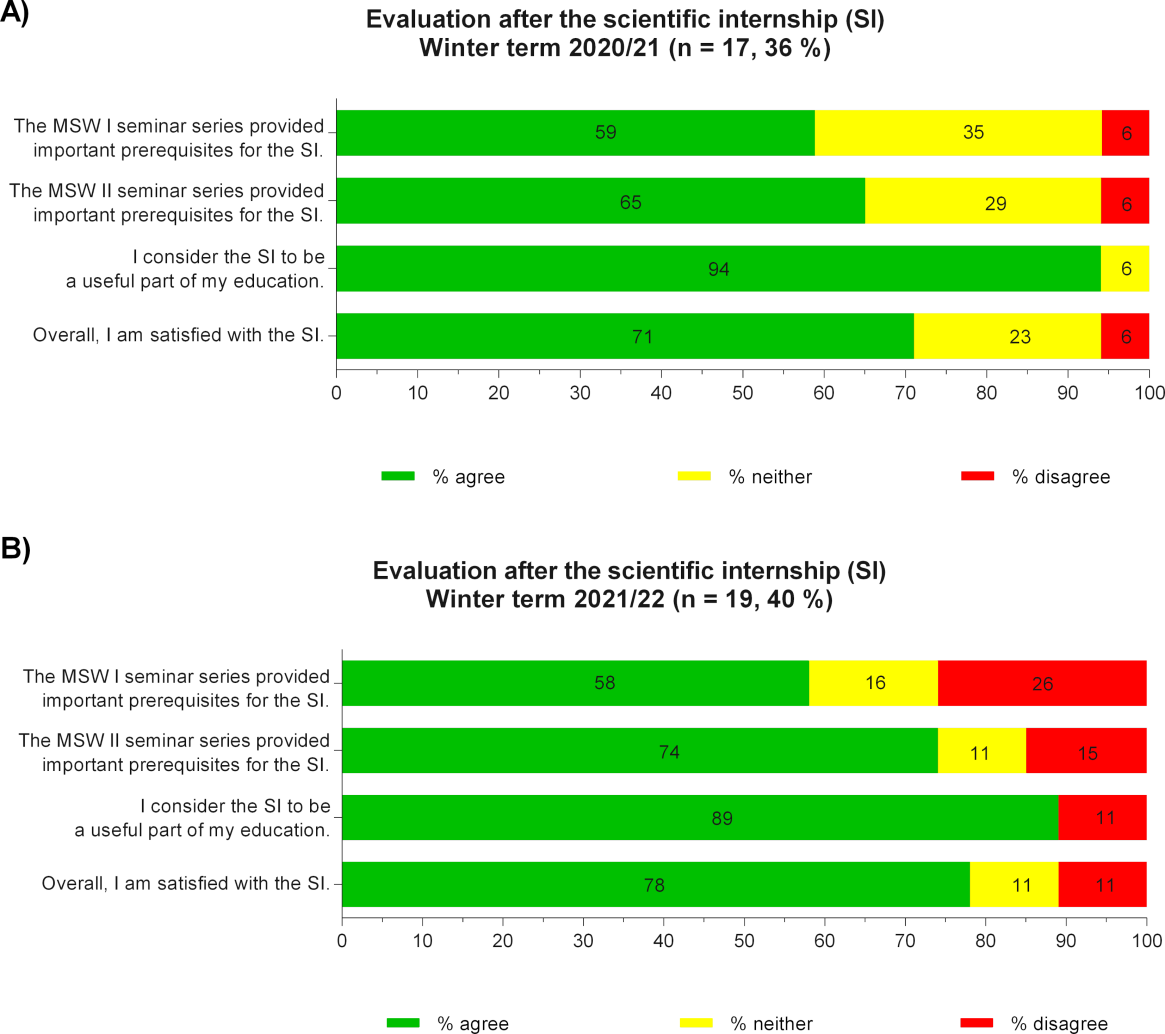
Evaluation results after complete revision of the Brandenburg Scientific Curriculum and completion of the Scientific Internship (SI) in winter 2020 (A) and winter 2021 (B)

Since winter 2020, the SI has been conducted for the first time on the basis of the complete BraWiC components MSW I & II and HS. In the student evaluation, an increase in the approval rates of MSW I was observed (see figure 3 (Fig. 3)). Thus, 59% and 58% of the participating students agreed that important prerequisites for the SI were taught. In addition, the rating of MSW II improved to 65% (1^st^ cohort) and 74% (2^nd^ cohort) respectively. As before, the SI received high approval rates (1^st^ cohort: 71%, 2^nd^ cohort: 78%) and was considered a useful educational experience (1^st^ cohort: 94%, 2^nd^ cohort: 89%). 

#### Comparison and mapping with NKLM LO

Of the NKLM chapter VIII.1 “Medical Scientific Skills”, 84 LOs (92.3%) could be located in the BMM, most of them in the BraWiC (n=55; 65.5%, see attachment 1 , table S1). A total of ten LOs (11.9%) could be located in other modules/lectures of the BMM (see attachment 1 , table S1). In addition, six LO were assigned to three other NKLM chapters (see attachment 1 , table S2), while one BraWiC LO could not be assigned to the NKLM. Due to the complexity of the MSW seminar series, see attachment 1 , table S3 provides a detailed listing of each course, semester sequence, and assigned LO.

## Discussion and lessons learned

A competent and reflective approach to a wide range of information, findings and new scientific knowledge is the cornerstone of good medical care. This must already be taught to students during their studies [8] and shall be specified by law through the amendment of the AO in Germany [9]. In it a mandatory scientific research internship of 480 TU and a longitudinal scientific curriculum is called for. The NKLM chapter VIII.1 “Medical and Scientific Skills” [https://nklm.de/zend/menu] can provide orientation in this regard. The longitudinal scientific curriculum of the BMM presented here can serve as an example for the concrete design. The BraWiC extends over semesters 1 to 10 (see figure 1 (Fig. 1)), comprises 474 TU and is composed of various modules and seminars, of which 320 TU are accounted for by the 8-week SI. As part of the AO amendment, this would only have to be extended by 4 weeks and would then already meet the requirements. The BraWiC could therefore serve as a blueprint that can be adapted to other medical faculties. However, as the medical sciences encompass the natural and social sciences as well as the humanities, the BraWiC also contains LO from other NKLM chapters. In addition, the BraWiC is linked to other teaching formats such as TRIK and problem-based learning as well as other modules of the BMM. This, we hope, enables students to adequately obtain the various skills necessary for scientifically-based clinical reasoning.

In addition to the discussion of key elements of curriculum development and corresponding assessment methods, the importance of faculty development for the successful implementation of a longitudinal scientific curriculum is outlined below. 

### Curriculum development and coordination

For the design, implementation and further development of the BraWiC it was essential to have a central coordination point. This included contact with all lecturers and coordination of learning content across semesters. This finding is supported by Strohmer et al. [31], who found similar factors in the implementation of a longitudinal communication curriculum. 

For the BraWiC, this was the responsibility of the Chair of Social Medicine and Epidemiology.

In designing the BraWiC, we deliberately chose the JC format to complement the problem-based, student-centred learning that is firmly anchored in the BMM, in order to promote independent and lifelong learning, and to apply previously acquired theoretical knowledge in the context of clinical-practical experience, despite other studies questioning the contribution of JC to the acquisition of EBM competences [32], [33], [34]. In doing so, the focus is on the discussion of the respective article for use in clinical practice, in addition to the critical evaluation of evidence. This is supported by the use of lecturer tandems, consisting of methodologists and clinicians from the respective disciplines, who focus on strengthening the student’s ability for clinical reasoning based on the critical reading of scientific studies.

### Development of assessment methods

Competency-based curricula require appropriate assessment methods that allow for verification of the targeted competencies and timely identification of the need to optimize the curriculum. As the development of competencies is a long-term process, it is particularly important to provide learners with regular feedback on their strengths and areas for improvement [35]. These formative assessments can help students proactively improve their performance on summative assessments [36]. 

Both formative and summative assessment formats have been introduced to evaluate the BraWiC milestones (see figure 1 (Fig. 1)). For example, the skills for planning a scientific research project are demonstrated by a project outline at the beginning of the 6^th^ semester. Students are required to formulate a concrete research question, as well as a scientific background and methodological considerations. They receive written feedback from the seminar instructors prior to the beginning of the SI.

The 2^nd^ milestone, the application of scientific basics to a specific research project, is assessed through summative assessment formats. These include a multiple-choice paper in ST, the written report of the SI, and a poster that is presented at the scientific poster congress at the end of the SI. 

The SI cover a broad spectrum of topics and disciplines. Projects and supervisors come from the natural and social sciences as well as the humanities. This diversity is a challenge for the assessment as disciplines adhere to different rules and have different epistemological approaches. Therefore, particular emphasis is placed on a coherent description of the study background, including the identification of the knowledge gap to be addressed, a clearly formulated research question, that structures the methods used in the study, and a reflection on the limitations and strengths of the study from the perspective of the respective discipline. These experiences are supported by Möller et al. [37], who also identified the formulation of a clear research question, the ability of students to write scientifically, and the feasibility of the study in a limited time as factors for successful SI. Through the accompanying MSW II seminars and poster presentations, students are exposed to the peculiarities of different disciplinary approaches and can experience how this diversity is the basis, strength, and challenge of the medical sciences.

The JC addresses the 3^rd^ milestone, the transfer of scientific thinking and action to the clinical practice, including strengthening scientific reasoning skills. Students evaluate current research literature and receive direct feedback through subsequent discussion with the accompanying seminar instructors. 

### Faculty development

Overall, the conception and implementation phase of scientific internships for a faculty should not be underestimated [20], [22]. In addition to the acquisition of sufficient and high-quality project offers as well as appropriately educated and trained supervisors, considerations regarding the project selection process, the possibilities of changing topics and/or supervisors, as well as counselling and support offers during the internship must also be taken into account. 

For the Brandenburg Medical School, it was also necessary to meet the challenges of a newly founded faculty, which is reflected both in the diversity of its teaching staff in terms of learning biographies, professional qualifications and previous teaching and research experience, as well as in its decentralised university structure. This requires consistent staff development to prepare supervisors for their new roles, but also to familiarise them with the curriculum [38]. For example, information sessions and materials, training on how to apply for ethics votes, and support and advice on how to develop student research projects have been and will continue to be offered to the teaching staff. In preparation for the poster congress, reviewers are trained and the conference itself serves as a communication platform within the university. 

### Limitations

The development of the BraWiC is based on existing (EBM-)curricula, the NKLM as well as on voluntary student evaluations of the first phase of the BraWiC. Particularly for the student evaluations some limitations need to be mentioned.

It is possible that those who are not content with the teaching or who are particularly content are more likely to evaluate than others. However, in the second cohort the participation was particularly high and results did not differ much from the first cohort. With regard to the evaluations after the curriculum revision, it should be noted that the participation rates of the respective cohorts were similar. However, it is not possible to say who the participating students were, so conversely, an overly positive impression may have been recorded. Finally, the written report of the SI is not evaluated independently from the supervisor. While this is common practice, it may lead to very different evaluations depending on the supervisor. 

## Conclusion

The BMM has implemented a scientific curriculum throughout the entire study programme, which is centrally coordinated and taught by lecturers from different disciplines, sometimes in mixed tandems of clinicians and methodologists. The aim is to train scientifically competent doctors who, in their later professional practice, will be able to search for, analyse and critically evaluate scientific research results for a specific problem, use them for clinical reasoning and clinical practice appropriately. In its complexity, the curriculum requires close and continuous coordination with all stakeholders, special consideration of methodological diversity in the preparation, implementation and evaluation of the SI, and comprehensive consideration of its potential for sustainable faculty development.

## Authors’ ORCIDs

- Julia Schendzielorz: [0000-0003-2471-094X]
- Christine Holmberg: [0000-0002-8852-4620]
- Philipp Jaehn: [0000-0002-1638-5158]

## Competing interests

The authors declare that they have no competing interests. 

## Supplementary Material

Supplementary tables

## Figures and Tables

**Table 1:**
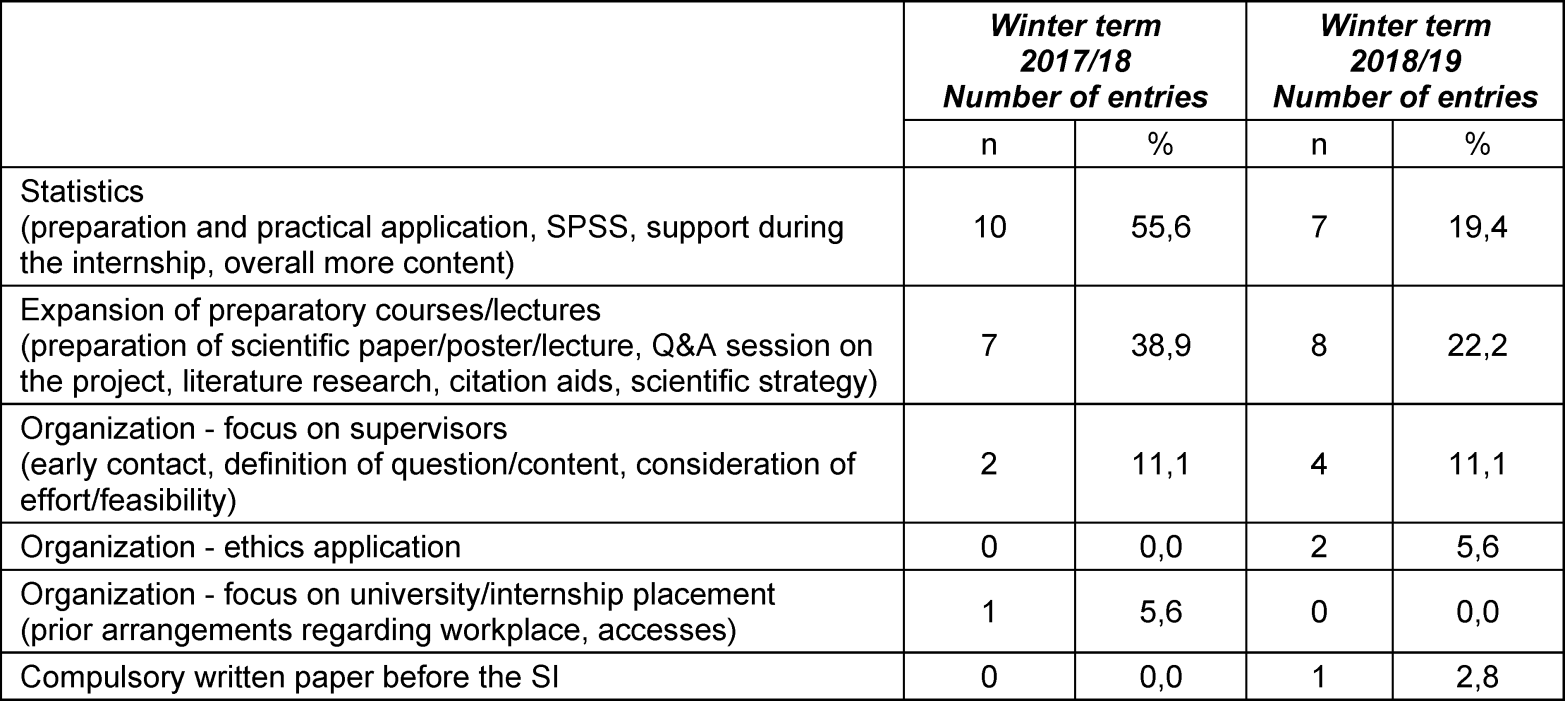
Categorization of the answers in relation to the question about suggestions for improvement
