# Supplementary material for: Planning, implementation and revision of the longitudinal scientific curriculum at the Medical School Brandenburg
Source: GMS J Med Educ. 2024 Apr 15;41(2):Doc16. doi: 10.3205/zma001671 (PMC11106572; doi:10.3205/zma001671)
Supplement: Supplementary tables [file JME-41-16-s-001.pdf]

Attachment 1: Supplementary tables

Attachment 1 to Schendzielorz J, Jaehn P, Holmberg C. *Planning, implementation and revision of the longitudinal scientific curriculum at the Medical School Brandenburg*. GMS J Med Educ. 2024;41(2):Doc16. DOI: 10.3205/zma001671

Attachment 1: Table S1

NKLM Chapter VIII.1: Medical-scientific skills

NKLM - ID	Competence / learning objective	Components of the BraWiC				Other modules of the BMM
		MSW	HS	ST	SI	
**VIII.1-01**	**Central concepts and theories of science: The graduates internalize the basics of scientific thinking and acting.**					
**VIII.1-01.1**	**They explain the basic concepts of medical research. They will be able to...**					
VIII.1-01.1.1	describe how causality is treated in medicine.	Movement				
VIII.1-01.1.2	use terms and methods relevant to medicine from different disciplines in a differentiated manner appropriate to scientific standards.	What is science?				GÄDH
**VIII.1-01.2**	**They apply basic principles of the philosophy of science and science research in the context of medicine. They will be able to...**					
VIII.1-01.2.1	distinguish scientific knowledge from other forms of knowledge.	What is science?<br>From the idea to the research question				
VIII.1-01.2.2	explain the historical development of experimentation, of animal and human experimentation, and the epistemology of medical research and reflect on one's own attitude against this background.	What is science?<br>Project presentations				HSS
VIII.1-01.2.3	discuss criteria of scientific rationality.	What is science?<br>Project presentations<br>Writing a scientific paper				
VIII.1-01.2.4	discuss the role of hypotheses in science.	Project outline: Status and questions<br>Project presentations				
VIII.1-01.2.5	characterize different concepts of medicine.					GÄDH
VIII.1-01.2.6	critically question the possibilities and limits of gaining medical knowledge.	Qualitative studies<br>From research to practice - Critical appraisal of RCTs				
VIII.1-01.2.7	delineate different science theory models for change in medical science and its related sciences.	What is science?				
VIII.1-01.2.8	classify the conditions of origin of the generation of scientific knowledge.	What is science?				HSS
VIII.1-01.2.9	analyze and reflect on knowledge of health and disease along social, biological, psychological, historical, and cultural dimensions.					NDM, Experience and Behavior, GÄDH
VIII.1-01.2.10	characterize medicine as a special form of knowledge in which scientific, practical and ethical knowledge are present in combination.	Introduction to medical guidelines				Healthcare
VIII.1-01.2.11	question the opportunities and limitations of scientific publishing.	Research career				
VIII.1-01.2.12	reflect on conflicts between roles as a physician and as a person acting scientifically.					Healthcare
**VIII.1-01.3**	**They align their scientific actions with the principles of good scientific practice. They will be able to...**					
VIII.1-01.3.1	explain the role of ethics committees in medical research.	Informed Consent, Ethics, Patient Safety & Ethics Committee I & II				
VIII.1-01.3.2	critically assess what level of collaboration justifies (co-)authorship in scientific publications.	Research career				
VIII.1-01.3.3	designate appropriate offices for suspected cases of scientific misconduct.	Good scientific practice, plagiarism and fabrication				
VIII.1-01.3.4	orient their actions to the goal of avoiding scientific misconduct.	Good scientific practice, plagiarism and fabrication				
VIII.1-01.3.5	assess the particular ethical challenges, legal frameworks and contexts of origin of research with human subjects (with special regard to vulnerable subjects) and populations in Germany and internationally.	Informed Consent, Ethics, Patient Safety & Ethics Committee I & II				HSS
VIII.1-01.3.6	be guided by the ethical and legal standards of good scientific practice.	Informed Consent, Ethics, Patient Safety & Ethics Committee I & II				

**VIII.1-02**	**Graduates review their professional knowledge and actions and continuously identify their own learning needs in terms of a lifelong learning process.**					
**VIII.1-02.1**	**They have mastered the principles of learning in the sense of recognizing and reflecting on their own learning needs and, derived from this, designing an adequate learning process and implementing learning outcomes. They will be able to...**					
VIII.1-02.1.1	Evaluate and reflect on the course as well as the outcome of a teaching-learning situation from the learner's point of view, also recognizing one's own limitations and abilities.	Time management<br>Introduction to scientific work (incl. learning types) I & II				TRIK<br>Mentoring*
VIII.1-02.1.2	formulate a specific (learning) question starting from a problem.	From the idea to the research question<br>From the research question to the project				PBL
VIII.1-02.1.3	evaluate their own level of training.	TRIK				Mentoring*, exams
VIII.1-02.1.4	search relevant (secondary and tertiary) literature and other sources of information using appropriate research systems and effective search strategies, make selections, and interpret.	Literature research I and II<br>Systematic literature review I and II<br>Structure and function of bibliographic databases<br>Literature management and citation				
VIII.1-02.1.5	keep abreast of new developments in content and changes in relevant framework conditions in medicine and health care and adapt their own medical knowledge and actions accordingly.	Introduction Critical Reading and Journal Club<br>Introduction to medical guidelines<br>Journal Club				
VIII.1-02.1.6	take responsibility for their continuing education to maintain and develop physician competencies by adequately assessing and evaluating their level of development in each competency area and taking appropriate action as necessary.					PBL, TRIK<br>Mentoring*, exams
**VIII.1-03**	**As critical users, the graduates explain the principles and methods of evidence-based medicine and apply them to problems in the treatment of individual patients and in the clinical context.**					
**VIII.1-03.1**	**They will be able to develop searchable questions based on clinical problems and conduct literature searches based on the best available evidence. They will be able to...**					
VIII.1-03.1.1	use relevant information technologies critically and in a patient-centered manner.					Clinical modules
VIII.1-03.1.2	translate patient-related problems into precise scientific questions that can be searched in specialist or literature databases.	Introduction to medical guidelines<br>Evidence-based Medicine<br>Journal Club				Clinical modules
VIII.1-03.1.3	identify and use multiple ways to obtain and manage literature.	Literature research I and II<br>Structure and function of bibliographic databases<br>Literature management and citation<br>Writing a scientific paper				PBL
VIII.1-03.1.4	formulate and conduct literature searches for the best available evidence for these problems using the precise, scientific questions in the databases relevant to one's profession.	Systematic literature review I and II<br>Structure and function of bibliographic databases				
**VIII.1-03.2**	**They will be able to critically develop an evaluation of the relevance and validity of the evidence found on a diagnostic problem. They will be able to...**					
VIII.1-03.2.1	explain the significance of the different types of diagnostic studies and discuss them in terms of their informative value for clinical application.	Cross-sectional and ecological studies<br>Case-control studies<br>Intervention studies<br>Journal Club				
VIII.1-03.2.2	discuss requirements of diagnostic studies for clinical use in a team setting.	Measurement error and questionnaire development<br>Journal Club				

VIII.1-03.2.3	conduct the review of a diagnostic study for its validity for clinical use.	Journal Club				
VIII.1-03.2.4	conduct the review of a diagnostic study with regard to its relevance for clinical application.	Journal Club				
**VIII.1-03.3**	**They will be able to critically develop an evaluation of the relevance and validity of the evidence found on a therapeutic problem. They will be able to...**					
VIII.1-03.3.1	discuss the characteristics and requirements of therapeutic and prognostic studies for clinical use.	Journal Club				
VIII.1-03.3.2	explain the characteristics of the different types of therapeutic and prognostic studies and discuss them with regard to their significance for clinical application.	Journal Club				
VIII.1-03.3.3	conduct the review of therapeutic and prognostic studies for their validity for clinical use.	Journal Club				
VIII.1-03.3.4	conduct the review of therapeutic and prognostic studies with regard to their relevance for clinical application.	Journal Club				Cardiovascular System, Healthcare
**VIII.1-03.4**	**They will be able to critically develop an evaluation of the relevance and validity of the evidence found in a systematic review. They will be able to...**					
VIII.1-03.4.1	explain the characteristics of the different study types of systematic reviews and discuss them in terms of their informative value for clinical application.	Systematic literature review I and II				
VIII.1-03.4.2	conduct the review of a systematic review with regard to its validity for clinical application.	Journal Club				
VIII.1-03.4.3	conduct the review of a systematic review with regard to its relevance for clinical application.	Journal Club				
VIII.1-03.4.4	discuss the characteristics and requirements of systematic reviews for clinical application.	Journal Club				
**VIII.1-03.5**	**They will be able to critically develop an evaluation of the relevance and validity of the evidence found in a guideline. They will be able to...**					
VIII.1-03.5.1	conduct a review of a guideline for its validity for clinical use.	Introduction to medical guidelines				
VIII.1-03.5.2	conduct the review of a guideline with regard to its relevance for clinical application.	Introduction to medical guidelines				
VIII.1-03.5.3	discuss the characteristics and requirements of clinical practice guidelines.	Study management<br>How to conduct lab research<br>Introduction to medical guidelines				
VIII.1-03.5.4	explain the characteristics of the different types of guideline studies and discuss them in terms of their informative value for clinical application.	Study management<br>How to conduct lab research<br>From research to practice - Critical appraisal of RCTs<br>Health Services Research				Healthcare
**VIII.1-03.6**	**They can present the evidence they have found and evaluated to patients in a form they can understand and integrate it into the treatment process. They will be able to...**					
VIII.1-03.6.1	articulate the validity and relevance of the evidence(s) assessed in a generally understandable manner using the appropriate communication model.					Healthcare, TRIK
VIII.1-03.6.2	discuss the results of the evaluated evidence(s) together with the patient in relation to his or her personal situation.					TRIK
VIII.1-03.6.3	integrate evidence collected and critically appraised for decision-making on a medical issue into the reality of care in a physician's daily practice.	Journal Club				Clinical modules
VIII.1-03.6.4	apply and discuss the methods of clinical decision making.	Introduction to medical guidelines<br>Evidence-based Medicine<br>Journal Club				CRDM
**VIII.1-03.7**	**They can plan their medical actions on the patient in an evidence-based manner and communicate in a patient-oriented manner. They will be able to...**					
VIII.1-03.7.1 (Practical	develop evidence-based treatment plans for individual patients in internal medicine, surgery, outpatients, and their elective and communicate them in a patient-friendly manner.					
VIII.1-03.7.2 (Practical Year)	develop evidence-based (differential) diagnostic work plans for individual patients in internal medicine, surgery, outpatients, and in their elective and communicate them in a patient-friendly manner.					
**VIII.1-03.8**	**They can explain and reflect on their own scientific medical approach to these problems. They will be able to...**					
VIII.1-03.8.1	present best available evidence to the team on a patient-centered aspect of care and evaluate it together at the organizational level for use in the physician's daily practice (Journal Club).	Journal Club				

VIII.1-03.8.2	formulate concrete advantages and disadvantages of science-based work in everyday medical practice and reflect on them on the basis of their own behavior.	Evidence-based Medicine<br>Possibilities and limits of shared decision making				CRDM
VIII.1-03.8.3	explain and discuss the principles and methods of evidence-based medicine and their application to problems in the treatment of individual patients as critical users.	Evidence-based Medicine<br>Possibilities and limits of shared decision making				Healthcare, CRDM
**VIII.1-04**	**The graduates contribute as innovator to the emergence, dissemination, application, and translation of new knowledge and practices.**					
**VIII.1-04.1**	**They use methodological skills in planning and evaluating scientific studies. They will be able to…**					
VIII.1-04.1.1	derive a scientifically answerable question.	From the idea to the research question				
VIII.1-04.1.2	explain the framework conditions for different types of studies.	Cross-sectional and ecological studies<br>Case-control studies<br>Qualitative studies<br>Participative research<br>Intervention studies<br>Study management				Healthcare
VIII.1-04.1.3	perceive one's own scientific specialization/limitation and, if necessary, obtain further research expertise.	Project outline: Status and questions<br>Project presentations<br>Statistical advice			**INT**	
VIII.1-04.1.4	present suitable study types for answering a research question and justify them in terms of their advantages and disadvantages.	Cross-sectional and ecological studies<br>Case-control studies<br>Qualitative studies<br>Intervention studies				
VIII.1-04.1.5	discuss different sampling techniques and justify their advantages and disadvantages.	Cross-sectional and ecological studies<br>Descriptive statistics<br>Qualitative studies				
VIII.1-04.1.6	justify the necessity of a case number estimate and name the prerequisites of a case number estimate.					
VIII.1-04.1.7	explain the basic principles of measurement in medical practice and research and apply them in the context of their own projects.	Measurement error and questionnaire development<br>Health-related quality of life as an endpoint				
VIII.1-04.1.8	justify the methods of scientifically sound data collection and transparent data management and use them appropriately depending on a project context.	Data management				
VIII.1-04.1.9	recognize a need for statistical consultation and, when consulting with a biometrician, present the information necessary for consultation in a qualified manner.	Statistical advice				
VIII.1-04.1.10	justify appropriate descriptive statistical methods and apply them to quantitative data collected.	Descriptive statistics<br>SPSS				
VIII.1-04.1.11	apply appropriate qualitative methods to qualitative data collected.	Qualitative studies				
VIII.1-04.1.12	interpret various textual, graphical, and tabular forms of presenting results and apply them in the context of their own projects.	Descriptive statistics<br>Journal Club				
VIII.1-04.1.13	apply statistical methods of hypothesis testing appropriately.					
VIII.1-04.1.14	apply methods of inferential statistics to determine the precision of estimates and interpret their results.					
VIII.1-04.1.15	describe simple regression methods and explain the scope and significance of the results.					
VIII.1-04.1.16	understand the meaning of confounding and outline techniques to control confounding.	Confounding und standardization				
VIII.1-04.1.17	understand causes and forms of bias and outline strategies to avoid bias.	Measurement error and questionnaire development<br>Confounding und standardization				

**VIII.1-04.2**	**They contribute to the emergence of new knowledge. They will be able to…**					
VIII.1-04.2.1	research, critically evaluate and summarize the current state of knowledge on a scientific issue.	Project outline: Status and questions				
VIII.1-04.2.2	apply the basic principles of project management to their research project.				**INT**	
VIII.1-04.2.3	critically discuss the significance of a scientific investigation with regard to methodological aspects.	Journal Club				
VIII.1-04.2.4	critically discuss results of an investigation in the context of existing evidence.					
VIII.1-04.2.5	critically discuss a gain in knowledge with regard to future research needs.					
VIII.1-04.2.6	prepare scientific results for a professional audience according to the rules of scientific publications.					
VIII.1-04.2.7	communicate own research results in an appropriate manner.					
**VIII.1-05**	**The graduates serve as teacher for various audiences (e.g., patients, students, others).**					
**VIII.1-05.1**	**They reflect on and evaluate teaching-learning situations in formal and informal professional contexts. They will be able to…**					
VIII.1-05.1.1	Evaluate adult education teaching, learning, and evaluation methods, as well as assessment, evaluation, and testing procedures, in terms of their strengths and weaknesses and the resources they require, and select them for a specific learning situation.					
VIII.1-05.1.2	assess the process of a teaching-learning situation from the perspective of the teacher, also reflecting on their own limits of knowledge and skills.					
VIII.1-05.1.3	provide learners with adequate feedback in specific situations.					TRIK
**VIII.1-05.2**	**They know the general principles of sustainable knowledge transfer and their specific application in the education, training and continuing education of medical professionals and members of other health care professions and apply them in their individual working environment. They will be able to...**					
VIII.1-05.2.1	identify the learning needs of the target group and select appropriate teaching content and formulate learning objectives.					PBL
VIII.1-05.2.2	select and apply appropriate teaching and evaluation methods according to the learning objectives and content.					PBL
**VIII.1-05.3**	**They will present and discuss the results of a scientific investigation. They will be able to…**					
VIII.1-05.3.1	present and critically discuss scientific results in an appropriate manner for a professional audience.	Journal Club				
**VIII.1-05.4**	**They know the general principles of sustainable knowledge transfer and their specific application in the education of patients, relatives and medical laypersons and apply them in their individual working environment. They will be able to…**					
VIII.1-05.4.1	identify information and learning needs according to the situation and select appropriate content and formulate communication goals.					Healthcare
VIII.1-05.4.2	select and apply appropriate and differentiated communication methods according to the communication objectives and content.					Healthcare, TRIK
**VIII.1-06**	**The graduates have competence in subject-specific scientific methods.**					
**VIII.1-06.1**	**They know methods from different areas of scientific research. They will be able to…**					
VIII.1-06.1.1	name possible objects of investigation and scientifically derive and justify their selection.	From the research question to the project<br>Project outline: Status and questions				
VIII.1-06.1.2	select exemplary investigation methods from two of four different areas on a scientific basis and carry them out practically (practical course 1 and 2).					
**VIII.1-07**	**The graduates will be able to conduct a scientific research paper.**					
**VIII.1-07.1**	**Within this framework, they will acquire scientific literacy in terms of topic identification, project planning, execution of the work, written documentation of the results, and presentation and discussion. They will be able to…**					
VIII.1-07.1.1	gain practical experience in a research project.				**INT**	
VIII.1-07.1.2	compose a written paper.	Writing a written report in the 1st semester; writing a project outline in the 5th semester				

BMM Brandenburg Reformed Medical Study Programme, BraWiC Brandenburg Scientific Curriculum, CRDM Clinical reasoning and decision making, GÄDH Principles of Medical Theory and Practice, HS Health Sciences, HSS Hormones/Sexual Organs/Sexuality, MSW Methods of Scientific Work, NDM Nutrition/ Digestion/Metabolism, NKLM National Competence Based Learning Objectives Catalogue Medicine, PBL Problem-based Learning, SI Scientific Internship, ST Statistics, TRIK Teamwork, Reflection, Interaction and Communication

Grey filled cells: Learning objective(s) could be assigned either to MSW, HS, SI and/or ST.

Dark grey filled cells: Learning objective(s) could not not be assigned to any component of the BraWiC/BMM. INT: Integration into the SI module is planned.

Mentoring* is in the process of being set up.

Attachment 1: Table S2

NKLM - ID	Learning objective - The graduates will be able to…	Components of the BraWiC			
		MSW	HS	ST	SI
**NKLM - Chapter VII.1 Principles of normal structure and function**					
VII.1a-20.2.1	explain the significance of demographic factors, social structures for health and illness, and health care.				
VII.1a-20.2.2	explain connections between social inequality and health and disease.				
**NKLM - Chapter VIII.4 Health counseling, promotion and prevention**					
VIII.4-03.2.2	name the concepts, models and variables of public health and global health.				
VIII.4-03.2.3	identify health policy goals and challenges at the national and international levels.				
VIII.4-03.8.2	understand epidemiologic measures and communicate them in language appropriate for the patient.				
**NKLM - Chapter VIII.6 Professional practice and ethics, history and law of medicine**					
VIII.6-01.2.7	treat patients respectfully while preserving their autonomy and orient their actions to their individual values and needs.				
**IMPP Objective Catalog - Graduate Profile: Scientific Activities - Planning and Implementation of a Research Project**					
N/A	The graduate is proficient in interdisciplinary teamwork while adhering to deadlines and communicating well.				

BraWiC Brandenburg Scientific Curriculum, IMPP Institute for medical and pharmaceutical examination questions, MSW Methods of Scientific Work, NKLM National Competence Based Learning Objectives Catalog Medicine, SI Scientific Internship, ST Statistics

Grey filled cells: Learning objective(s) could be assigned either to HS, SI and/or ST.

Attachment 1: Table S3

Longitudinal Seminar Series Methods of Scientific Work - Learning Objective Assignments

Semester	Topic / thematic focus	TU	NKLM	BMM
1	What is science?	2	VIII.1-01.1.2, VIII.1-01.2.1/2/3, VIII.1-01.2.7/8	
1	Literature research I and II	4	VIII.1-02.1.4, VIII.1-03.1.3	
1	Time management	2	VIII.1-02.1.1	
1	Introduction to scientific work (incl. learning types) I & II	6	VIII.1-02.1.1, VIII.1-07.1.2	
3	Measurement error and questionnaire development	2	VIII.1-03.2.2, VIII.1-04.1.7, VIII.1-04.1.17	
3	Cross-sectional and ecological studies	2	VIII.1-03.2.1, VIII.1-04.1.2, VIII.1-04.1.4/5	
3	Case-control studies	2	VIII.1-03.2.1, VIII.1-04.1.2, VIII.1-04.1.4	
3	Descriptive statistics	2	VIII.1-04.1.5, VIII.1-04.1.10, VIII.1-04.1.12	
4	Qualitative studies	2	VIII.1-01.1.1, VIII.1-01.2.6, VIII.1-04.1.2, VIII.1-04.1.4/5, VIII.1-04.1.11	
4	Participative research	2	VIII.1-04.1.2	
4	Confounding and standardization	2	VIII.1-01.1.1, VIII.1-04.1.16/17	
4	Intervention studies	2	VIII.1-01.1.1, VIII.1-03.2.1, VIII.1-04.1.2, VIII.1-04.1.4	
4	Systematic literature review I and II	4	VIII.1-02.1.4, VIII.1-03.1.4, VIII.1-03.4.1; VIII.1-03.4.4	
5	Study management	2	VIII.1-03.5.3/4, VIII.1-04.1.2	
5	Structure and function of bibliographic databases	2	VIII.1-02.1.4, VIII.1-03.1.3/4	
5	Good scientific practice, plagiarism and fabrication	2	VIII.1-01.3.3/4	
5	Medical Scientific English	2		1,2
5	Informed Consent, Ethics, Patient Safety & Ethics Committee I & II	4	VIII.1-01.3.1, VIII.1-01.3.5/6	
5	From the idea to the research question	2	VIII.1-01.2.1/2, VIII.1-04.1.1	
5	From the research question to the project	2	VIII.1-02.1.2, VIII.1-06.1.1	
	**MSW I - Total TU**	**50**		
6	Project outline: Status and questions	2	VIII.1-01.2.4, VIII.1-04.1.3, VIII.1-06.1.1, VIII.1-07.1.2	
6	Literature management and citation	2	VIII.1-02.1.4, VIII.1-03.1.3	
6	How to conduct lab research	2	VIII.1-03.5.3/4, VIII.1-04.1.2	
6	Data management	2	VIII.1-04.1.8	
6	Preclinical and clinical research - from bench to bedside	2		3
6	Health-related quality of life as an endpoint	2	VIII.1-04.1.7	
6	Health Services Research	2	VIII.1-04.1.2, VIII.1-03.5.4	
6	Research career	2	VIII.1-01.2.11, VIII.1-01.3.2	
6	Writing a scientific paper	2	VIII.1-01.2.3, VIII.1-03.1.3, VIII.1-05.3.1	
6	Creating a scientific poster	2	VIII.1-05.3.1	
6	Project presentations	2	VIII.1-01.2.2/3/4, VIII.1-04.1.3	
6	SPSS	4	VIII.1-04.1.10	
6	Statistical advice	2	VIII.1-04.1.3, VIII.1-04.1.9	
	**MSW II - Total TU**	**28**		
7	Introduction Critical Reading and Journal Club	2	VIII.1-02.1.5	
7	Introduction to medical guidelines	2	VIII.1-01.2.10, VIII.1-02.1.5, VIII.1-03.1.2, VIII.1-03.4.1, VIII.1-03.4.4, VIII.1-03.5.1/2/3, VIII.1-03.6.4	
7	From research to practice - Critical appraisal of RCTs	2	VIII.1-01.2.6, VIII.1-03.4.1, VIII.1-03.5.4	
7	Evidence-Based Medicine	2	VIII.1-03.1.2, VIII.1-03.6.4, VIII.1-03.8.2/3	
7	Possibilities and limits of shared decision making	2	VIII.1-03.8.2/3	
7	Register Research	2	VIII.1-04.1.2	
8	Journal Club - Gynecology I & II	4		
8	Journal Club - Pediatrics I & II	4		
9	Journal Club - Neurology I	2		
9	Journal Club - Psychiatry I	2	VIII.1-02.1.5, VIII.1-03.1.2, VIII.1-03.2./3/4, **VIII.1-03.3.1/2/3/4**, VIII.1-03.4.2/3/4, VIII.1-03.6.3/4, VIII.1-03.8.1, VIII.1-04.1.12, VIII.1-04.2.3, VIII.1-05.3.1	
9	Journal Club - Surgery I & II	4		
10	Journal Club - Medicine I & II	4		
10	Journal Club - Elderly Care I & II	4		
	**MSW III - Total TU**	**36**		

BMM Brandenburg Reformed Medical Study Programme, MSW Methods of Scientific Work, NKLM National Competence Based Learning Objectives Catalogue Medicine, TU Teaching unit

Learning Objective 1: Be able to describe the basic characteristics of English science language and texts and key differences from German.

Learning Objective 2: Be able to apply various techniques for reading English science texts.

Learning Objective 3: Be able to reflect on how basic research, clinical research, and implementation in the clinic relate to each other.

Bold print: Focus on therapeutic studies.
